# Ensembl 2026

**DOI:** 10.1093/nar/gkaf1239

**Published:** 2025-11-26

**Authors:** Andrew D Yates, Olanrewaju Austine-Orimoloye, Andrey G Azov, Matthieu Barba, If Barnes, Vianey Paola Barrera-Enriquez, Arne Becker, Ruth Bennett, Andrew Berry, Jyothish Bhai, Simarpreet Kaur Bhurji, Paulo R Branco Lins, Lucy Brooks, Shashank Budhanuru Ramaraju, Lahcen I Campbell, Manuel Carbajo Martinez, Jack Carpenter, Mehrnaz Charkhchi, Lucas A Cortes, Claire Davidson, Suzanna Dickson, Kamalkumar Dodiya, Sarah Donaldson, Bilal El Houdaigui, Tamara El Naboulsi, Aine Fairbrother-Browne, Oluwadamilare Falola, Reham Fatima, Jose Gonzalez Martinez, Tatiana Gurbich, Holly Hall, Matthew Hardy, Zoe Hollis, Toby Hunt, Mike Kay, Vinay Kaikala, Anna Lazar, Diana Lemos, Disha Lodha, Nourhen Mathlouthi, Gabriela A Merino, Ryan Merritt, Louisse Paola Mirabueno, Aleena Mushtaq, Syed Nakib Hossain, José G Pérez-Silva, Ivana Piližota, Daniel Poppleton, Irina Prosovetskaia, Shriya Raj, Ahamed Imran Abdul Salam, Shradha Saraf, Swati Sinha, Botond Sipos, Vasily Sitnik, Marie-Marthe Suner, Likhitha Surapaneni, Jack A S Tierney, David Urbina-Gómez, Andres Veidenberg, Thomas A Walsh, Jamie M Allen, Jorge Alvarez-Jarreta, Jitender Cheema, Jorge Batista da Rocha, Nishadi H De Silva, Francesca Floriana Tricomi, Stefano Giorgetti, Garth R Ilsley, Jon Keatley, Jane E Loveland, Jonathan M Mudge, Guy Naamati, John Tate, Natalie L Willhoft, Andrea Winterbottom, Bethany R Flint, Adam Frankish, Leanne Haggerty, Sarah E Hunt, Emily L Clark, Sarah C Dyer, Mallory A Freeberg, Fergal J Martin, Robert D Finn

**Affiliations:** European Molecular Biology Laboratory, European Bioinformatics Institute, Wellcome Genome Campus, Hinxton, Cambridge CB10 1SD, United Kingdom; European Molecular Biology Laboratory, European Bioinformatics Institute, Wellcome Genome Campus, Hinxton, Cambridge CB10 1SD, United Kingdom; European Molecular Biology Laboratory, European Bioinformatics Institute, Wellcome Genome Campus, Hinxton, Cambridge CB10 1SD, United Kingdom; European Molecular Biology Laboratory, European Bioinformatics Institute, Wellcome Genome Campus, Hinxton, Cambridge CB10 1SD, United Kingdom; European Molecular Biology Laboratory, European Bioinformatics Institute, Wellcome Genome Campus, Hinxton, Cambridge CB10 1SD, United Kingdom; European Molecular Biology Laboratory, European Bioinformatics Institute, Wellcome Genome Campus, Hinxton, Cambridge CB10 1SD, United Kingdom; European Molecular Biology Laboratory, European Bioinformatics Institute, Wellcome Genome Campus, Hinxton, Cambridge CB10 1SD, United Kingdom; European Molecular Biology Laboratory, European Bioinformatics Institute, Wellcome Genome Campus, Hinxton, Cambridge CB10 1SD, United Kingdom; European Molecular Biology Laboratory, European Bioinformatics Institute, Wellcome Genome Campus, Hinxton, Cambridge CB10 1SD, United Kingdom; European Molecular Biology Laboratory, European Bioinformatics Institute, Wellcome Genome Campus, Hinxton, Cambridge CB10 1SD, United Kingdom; European Molecular Biology Laboratory, European Bioinformatics Institute, Wellcome Genome Campus, Hinxton, Cambridge CB10 1SD, United Kingdom; European Molecular Biology Laboratory, European Bioinformatics Institute, Wellcome Genome Campus, Hinxton, Cambridge CB10 1SD, United Kingdom; European Molecular Biology Laboratory, European Bioinformatics Institute, Wellcome Genome Campus, Hinxton, Cambridge CB10 1SD, United Kingdom; European Molecular Biology Laboratory, European Bioinformatics Institute, Wellcome Genome Campus, Hinxton, Cambridge CB10 1SD, United Kingdom; European Molecular Biology Laboratory, European Bioinformatics Institute, Wellcome Genome Campus, Hinxton, Cambridge CB10 1SD, United Kingdom; European Molecular Biology Laboratory, European Bioinformatics Institute, Wellcome Genome Campus, Hinxton, Cambridge CB10 1SD, United Kingdom; European Molecular Biology Laboratory, European Bioinformatics Institute, Wellcome Genome Campus, Hinxton, Cambridge CB10 1SD, United Kingdom; School of Biosciences, Cardiff University, Cardiff CF10 3AX, United Kingdom; European Molecular Biology Laboratory, European Bioinformatics Institute, Wellcome Genome Campus, Hinxton, Cambridge CB10 1SD, United Kingdom; European Molecular Biology Laboratory, European Bioinformatics Institute, Wellcome Genome Campus, Hinxton, Cambridge CB10 1SD, United Kingdom; Faculty of Medical Sciences, Newcastle University, Newcastle upon TyneNE1 7RU,United Kingdom; European Molecular Biology Laboratory, European Bioinformatics Institute, Wellcome Genome Campus, Hinxton, Cambridge CB10 1SD, United Kingdom; European Molecular Biology Laboratory, European Bioinformatics Institute, Wellcome Genome Campus, Hinxton, Cambridge CB10 1SD, United Kingdom; School of Life Sciences, University of Warwick, CoventryCV4 7AL, United Kingdom; European Molecular Biology Laboratory, European Bioinformatics Institute, Wellcome Genome Campus, Hinxton, Cambridge CB10 1SD, United Kingdom; European Molecular Biology Laboratory, European Bioinformatics Institute, Wellcome Genome Campus, Hinxton, Cambridge CB10 1SD, United Kingdom; European Molecular Biology Laboratory, European Bioinformatics Institute, Wellcome Genome Campus, Hinxton, Cambridge CB10 1SD, United Kingdom; European Molecular Biology Laboratory, European Bioinformatics Institute, Wellcome Genome Campus, Hinxton, Cambridge CB10 1SD, United Kingdom; European Molecular Biology Laboratory, European Bioinformatics Institute, Wellcome Genome Campus, Hinxton, Cambridge CB10 1SD, United Kingdom; European Molecular Biology Laboratory, European Bioinformatics Institute, Wellcome Genome Campus, Hinxton, Cambridge CB10 1SD, United Kingdom; European Molecular Biology Laboratory, European Bioinformatics Institute, Wellcome Genome Campus, Hinxton, Cambridge CB10 1SD, United Kingdom; European Molecular Biology Laboratory, European Bioinformatics Institute, Wellcome Genome Campus, Hinxton, Cambridge CB10 1SD, United Kingdom; European Molecular Biology Laboratory, European Bioinformatics Institute, Wellcome Genome Campus, Hinxton, Cambridge CB10 1SD, United Kingdom; European Molecular Biology Laboratory, European Bioinformatics Institute, Wellcome Genome Campus, Hinxton, Cambridge CB10 1SD, United Kingdom; European Molecular Biology Laboratory, European Bioinformatics Institute, Wellcome Genome Campus, Hinxton, Cambridge CB10 1SD, United Kingdom; European Molecular Biology Laboratory, European Bioinformatics Institute, Wellcome Genome Campus, Hinxton, Cambridge CB10 1SD, United Kingdom; European Molecular Biology Laboratory, European Bioinformatics Institute, Wellcome Genome Campus, Hinxton, Cambridge CB10 1SD, United Kingdom; European Molecular Biology Laboratory, European Bioinformatics Institute, Wellcome Genome Campus, Hinxton, Cambridge CB10 1SD, United Kingdom; European Molecular Biology Laboratory, European Bioinformatics Institute, Wellcome Genome Campus, Hinxton, Cambridge CB10 1SD, United Kingdom; European Molecular Biology Laboratory, European Bioinformatics Institute, Wellcome Genome Campus, Hinxton, Cambridge CB10 1SD, United Kingdom; European Molecular Biology Laboratory, European Bioinformatics Institute, Wellcome Genome Campus, Hinxton, Cambridge CB10 1SD, United Kingdom; European Molecular Biology Laboratory, European Bioinformatics Institute, Wellcome Genome Campus, Hinxton, Cambridge CB10 1SD, United Kingdom; European Molecular Biology Laboratory, European Bioinformatics Institute, Wellcome Genome Campus, Hinxton, Cambridge CB10 1SD, United Kingdom; European Molecular Biology Laboratory, European Bioinformatics Institute, Wellcome Genome Campus, Hinxton, Cambridge CB10 1SD, United Kingdom; European Molecular Biology Laboratory, European Bioinformatics Institute, Wellcome Genome Campus, Hinxton, Cambridge CB10 1SD, United Kingdom; European Molecular Biology Laboratory, European Bioinformatics Institute, Wellcome Genome Campus, Hinxton, Cambridge CB10 1SD, United Kingdom; European Molecular Biology Laboratory, European Bioinformatics Institute, Wellcome Genome Campus, Hinxton, Cambridge CB10 1SD, United Kingdom; European Molecular Biology Laboratory, European Bioinformatics Institute, Wellcome Genome Campus, Hinxton, Cambridge CB10 1SD, United Kingdom; European Molecular Biology Laboratory, European Bioinformatics Institute, Wellcome Genome Campus, Hinxton, Cambridge CB10 1SD, United Kingdom; European Molecular Biology Laboratory, European Bioinformatics Institute, Wellcome Genome Campus, Hinxton, Cambridge CB10 1SD, United Kingdom; European Molecular Biology Laboratory, European Bioinformatics Institute, Wellcome Genome Campus, Hinxton, Cambridge CB10 1SD, United Kingdom; European Molecular Biology Laboratory, European Bioinformatics Institute, Wellcome Genome Campus, Hinxton, Cambridge CB10 1SD, United Kingdom; European Molecular Biology Laboratory, European Bioinformatics Institute, Wellcome Genome Campus, Hinxton, Cambridge CB10 1SD, United Kingdom; European Molecular Biology Laboratory, European Bioinformatics Institute, Wellcome Genome Campus, Hinxton, Cambridge CB10 1SD, United Kingdom; European Molecular Biology Laboratory, European Bioinformatics Institute, Wellcome Genome Campus, Hinxton, Cambridge CB10 1SD, United Kingdom; European Molecular Biology Laboratory, European Bioinformatics Institute, Wellcome Genome Campus, Hinxton, Cambridge CB10 1SD, United Kingdom; European Molecular Biology Laboratory, European Bioinformatics Institute, Wellcome Genome Campus, Hinxton, Cambridge CB10 1SD, United Kingdom; European Molecular Biology Laboratory, European Bioinformatics Institute, Wellcome Genome Campus, Hinxton, Cambridge CB10 1SD, United Kingdom; European Molecular Biology Laboratory, European Bioinformatics Institute, Wellcome Genome Campus, Hinxton, Cambridge CB10 1SD, United Kingdom; European Molecular Biology Laboratory, European Bioinformatics Institute, Wellcome Genome Campus, Hinxton, Cambridge CB10 1SD, United Kingdom; European Molecular Biology Laboratory, European Bioinformatics Institute, Wellcome Genome Campus, Hinxton, Cambridge CB10 1SD, United Kingdom; European Molecular Biology Laboratory, European Bioinformatics Institute, Wellcome Genome Campus, Hinxton, Cambridge CB10 1SD, United Kingdom; European Molecular Biology Laboratory, European Bioinformatics Institute, Wellcome Genome Campus, Hinxton, Cambridge CB10 1SD, United Kingdom; European Molecular Biology Laboratory, European Bioinformatics Institute, Wellcome Genome Campus, Hinxton, Cambridge CB10 1SD, United Kingdom; European Molecular Biology Laboratory, European Bioinformatics Institute, Wellcome Genome Campus, Hinxton, Cambridge CB10 1SD, United Kingdom; European Molecular Biology Laboratory, European Bioinformatics Institute, Wellcome Genome Campus, Hinxton, Cambridge CB10 1SD, United Kingdom; European Molecular Biology Laboratory, European Bioinformatics Institute, Wellcome Genome Campus, Hinxton, Cambridge CB10 1SD, United Kingdom; European Molecular Biology Laboratory, European Bioinformatics Institute, Wellcome Genome Campus, Hinxton, Cambridge CB10 1SD, United Kingdom; European Molecular Biology Laboratory, European Bioinformatics Institute, Wellcome Genome Campus, Hinxton, Cambridge CB10 1SD, United Kingdom; European Molecular Biology Laboratory, European Bioinformatics Institute, Wellcome Genome Campus, Hinxton, Cambridge CB10 1SD, United Kingdom; European Molecular Biology Laboratory, European Bioinformatics Institute, Wellcome Genome Campus, Hinxton, Cambridge CB10 1SD, United Kingdom; European Molecular Biology Laboratory, European Bioinformatics Institute, Wellcome Genome Campus, Hinxton, Cambridge CB10 1SD, United Kingdom; European Molecular Biology Laboratory, European Bioinformatics Institute, Wellcome Genome Campus, Hinxton, Cambridge CB10 1SD, United Kingdom; European Molecular Biology Laboratory, European Bioinformatics Institute, Wellcome Genome Campus, Hinxton, Cambridge CB10 1SD, United Kingdom; European Molecular Biology Laboratory, European Bioinformatics Institute, Wellcome Genome Campus, Hinxton, Cambridge CB10 1SD, United Kingdom; European Molecular Biology Laboratory, European Bioinformatics Institute, Wellcome Genome Campus, Hinxton, Cambridge CB10 1SD, United Kingdom; European Molecular Biology Laboratory, European Bioinformatics Institute, Wellcome Genome Campus, Hinxton, Cambridge CB10 1SD, United Kingdom; European Molecular Biology Laboratory, European Bioinformatics Institute, Wellcome Genome Campus, Hinxton, Cambridge CB10 1SD, United Kingdom; European Molecular Biology Laboratory, European Bioinformatics Institute, Wellcome Genome Campus, Hinxton, Cambridge CB10 1SD, United Kingdom; European Molecular Biology Laboratory, European Bioinformatics Institute, Wellcome Genome Campus, Hinxton, Cambridge CB10 1SD, United Kingdom; European Molecular Biology Laboratory, European Bioinformatics Institute, Wellcome Genome Campus, Hinxton, Cambridge CB10 1SD, United Kingdom; European Molecular Biology Laboratory, European Bioinformatics Institute, Wellcome Genome Campus, Hinxton, Cambridge CB10 1SD, United Kingdom; European Molecular Biology Laboratory, European Bioinformatics Institute, Wellcome Genome Campus, Hinxton, Cambridge CB10 1SD, United Kingdom; European Molecular Biology Laboratory, European Bioinformatics Institute, Wellcome Genome Campus, Hinxton, Cambridge CB10 1SD, United Kingdom; European Molecular Biology Laboratory, European Bioinformatics Institute, Wellcome Genome Campus, Hinxton, Cambridge CB10 1SD, United Kingdom; European Molecular Biology Laboratory, European Bioinformatics Institute, Wellcome Genome Campus, Hinxton, Cambridge CB10 1SD, United Kingdom; European Molecular Biology Laboratory, European Bioinformatics Institute, Wellcome Genome Campus, Hinxton, Cambridge CB10 1SD, United Kingdom; European Molecular Biology Laboratory, European Bioinformatics Institute, Wellcome Genome Campus, Hinxton, Cambridge CB10 1SD, United Kingdom

## Abstract

The Ensembl project (https://www.ensembl.org) is a public and open resource providing access to genomes, annotations, high-quality tools, and methods applicable to species from across the tree of life. This year has witnessed nearly a doubling in our rate of annotation and genome release, with 1927 new genomes released, with the total number of genomes now standing at 37 546. This includes expanded support for the human and barley pangenomes. We also present two new interfaces providing improved mechanisms to explore and interrogate genome regulation annotations. As our focus remains on sustainable scaling, we have archived Ensembl Rapid Release and accelerated the move to the new Ensembl platform. Ensembl release 116 (Q1-2026) will be the last release on the current platform.

## Graphical Abstract

**Graphical Abstract:**
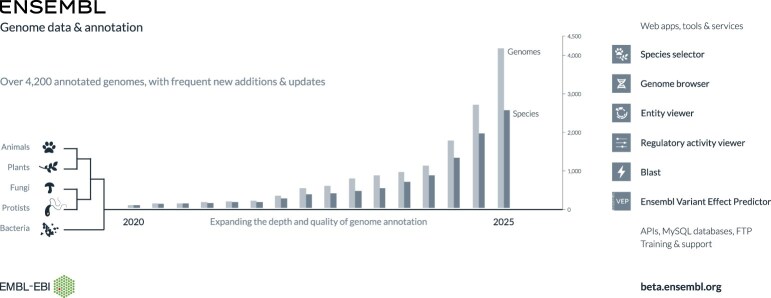

## Introduction

For 26 years, the Ensembl project (www.ensembl.org) has been providing high-quality reference genome annotation across the taxonomic space. Originating from the human genome project, the resource has grown to provide support for model organisms, vertebrates, microbes, plants, fungi, and other metazoans producing one of the world's most comprehensive genomic annotation resources. We annotate genes and transcripts, small- and large-scale genetic variation, genome regulatory elements, and comparative genomics (orthology and whole-genome alignments). These data are produced by our open, in-house analysis methods and supplemented with other annotation providers such as RefSeq and VEuPathDB [1, 2]. All genome sequences must be submitted to the International Nucleotide Sequence Database Collaboration and be publicly available as required by a joint browser agreement with the National Center for Biotechnology Information (NCBI) and the University of California, Santa Cruz (UCSC) Genome Browser [3–5]. Ensembl is an ELIXIR Core Data Resource and a Global Core Biodata Resource, highlighting the centrality of the resource to global research.

Ensembl data are available in standard file formats, without restrictions, through our websites, BioMart, REST application programming interfaces (APIs) (https://rest.ensembl.org), MySQL databases, and FTP site [6, 7]. Ensembl software is available from GitHub (https://github.com/Ensembl and https://github.com/EnsemblGenomes) including the Ensembl Variant Effect Predictor (Ensembl VEP) [8]. All Ensembl data are made available under the EMBL-EBI Terms of Use and software under an Apache 2.0 license

Ensembl Beta, our unified platform for accessing genomes across the tree of life, houses both newly generated datasets and the majority of data hosted in our dedicated portals: Vertebrates, Plants (plants.ensembl.org), Metazoa (metazoa.ensembl.org), Fungi (fungi.ensembl.org), Protists (protists.ensembl.org), and GRCh37 (grch37.ensembl.org). Data hosted in our Bacteria (bacteria.ensembl.org) and SARS-CoV-2 (covid-19.ensembl.org) portals will migrate during 2026. Our new platform receives updates approximately every two weeks and provides access to over 4200 genomes. Ensembl releases will continue on the current platform until release 116 (Q1-2026), after which all future data releases will be made through our new platform.

### Broadening genome annotation resources

Ensembl continues to support an expanding community through increasing coverage of biodiversity projects including Darwin Tree of Life, European Reference Genome Atlas, Canada Biogenome Project, Aquatic Symbiosis Genomics Project, and Earth BioGenome Project (EBP) [9–12]. Over the past year, we have released 1927 new genomes representing 946 unique species across 20 phyla. New barley, oat, grape, pea, and lablab bean assemblies are available alongside large-scale variation for wheat and rice [13–18]. Livestock, companion animals, and rodents have received updates for sheep (new breed and reference) and cattle (new breeds and regulation annotation), cat, and rat, respectively (Table 1) [19–21]. We have imported new assemblies for key disease vectors and pests alongside alignment of genomes with VEuPathDB. All genomes made available from Ensembl Beta have homology predictions based on reciprocal best BLAST hits to align genomes with one of 11 pre-defined taxonomic collections, e.g. *Liliopsida* or *Mammalia*, or with a default set of 36 representative genomes if a specific group is not applicable [22].

**Table 1.** Summary of new assemblies, annotations, and datasets across plants, animals, insects, and microbes

Domain	Species/group	Update type
Companion animals	*Felis catus*	Assembly update (Fca126_mat1.0)
Crops	*Avena* spp. (oats)	4 new assemblies
Crops	*Hordeum vulgare* (barley)	75 cultivars, pangenome expansion, multiple whole-genome alignment (WGA) and gene trees
Crops	*Oryza sativa* (rice)	Variation from 3024 accessions (3K project)
Crops	*Pisum sativum* (pea), *S. stenocarpa* (African yam bean), *L. purpureus* (lablab bean)	New assemblies
Crops	*Triticum aestivum* (wheat)	New cultivar (Alchemy); variation from TaNG SNP and Watkins Core
Crops	*Vitis vinifera* (grape)	PN40024 telomere-to-telomere assembly
Disease vectors	*Amblyomma americanum* (lone star tick), *Ornithodoros turicata* (softbacked tick)	New assemblies
Fungi/Protists	*Plasmodium falciparum, Fusarium graminearum*	24 genomes imported from VEuPathDB
Insects	*Drosophila* spp.	6 new assemblies and pangenome WGA
Livestock	*Bos taurus* (cattle)	2 new breeds; ARS-UCD2.0 regulatory data (functional annotation of animal genomes)
Livestock	*Ovis aries* (sheep)	New breed annotations; Rambouillet update incl. Y chr.
Livestock	*Sus scrofa* (pigs)	Pangenome WGA update
Pests	*Vespa mandarinia* (Asian giant hornet)*, Bactrocera oleae* (olive fruit fly)*, Citripestis eutraphera* (mango seed moth)	New assemblies
Rodents	*Mus musculus*	Mouse strains WGA update
Rodents	*Rattus norvegicus*	Assembly update (GRCr8)

To meet challenges in structurally annotating genes from genomes lacking transcriptomic data, we have adopted two new machine learning and hidden Markov model-based tools: Helixer and Tiberius [23, 24]. These methods model gene structure directly from a genomic sequence and are applicable across a wide taxonomic spread with minimal parameter tuning, in contrast to BRAKER2, which relies on species-specific training data and external homology evidence. *Umbilicaria deusta* (GCA_964340765.1) is our first released annotation based on Helixer. To help differentiate between methods, we include the annotation methodology as part of dataset metadata available from the Ensembl Beta website. These two new methods supersede BRAKER2 as our default annotation system for such cases [25].

### Human annotation

The integration of long-read transcriptomic data has led to a significant increase in annotation of full-length protein-coding transcripts in GRCh38. Ensembl 115 contained ∼121 000 new protein-coding transcripts (a 2.4-fold increase) added to the GRCh38 human reference gene set via the GENCODE TAGENE pipeline [26, 27]. This increase necessitated the use of our previously described GENCODE Primary subset methodology to identify and rank transcripts with high functional potential through expression and evolutionary constraint data [28]. Since Ensembl 114, GENCODE Primary is the default annotation for both human GRCh38 and mouse GRCm39 in our analysis and visualization tools, including Ensembl VEP, and is flagged in our GFF3 files [28]. GENCODE Primary enables faster downstream analysis by excluding exons and splice sites that lack evidence of evolutionary conservation or constraint or with low expression/inclusion. Additionally, the Matched Annotation from NCBI and EMBL-EBI (MANE) collaborative dataset has been updated to version 1.4 [29] and contains 50 disease-associated non-coding genes, including RNU4ATAC, which is implicated in RNU4ATAC Spectrum Disorder [30]. The Ensembl Transcript Archive (Tark) has been updated to include these collective annotation updates. Continuing our support of human pangenomics, we have annotated the second release of the Human Pangenome Reference Consortium representing the largest public set of assembled human genomes from 232 individuals of diverse ancestries and 464 haploid genomes annotated using projection from GENCODE 47 [31].

We continue to update variation and phenotype resources, providing a comprehensive description of human variation integrating data from dbSNP, gnomAD, NHGRI-EBI GWAS Catalog, COSMIC, and UniProt [32–35]. Phenotype association pages now display clinical impact classifications for somatic variants from ClinVar, enhancing support for cancer variant interpretation. GRCh37 and CHM13-T2T have been updated with population allele frequencies from gnomAD v4.1.

GRCh38 regulatory feature annotation has been refined by incorporating additional open chromatin and histone ChIP-seq data from the Encyclopedia of DNA Elements (ENCODE), with promoter annotation updated to match gene annotation from GENCODE 48. This led to a small increase in total features from 370 183 to 380 818, but notably a reduction in unclassified open chromatin regions from 19 029 to 7541, with most now classified as enhancers. Both motif features and regulatory annotation from GRCh38 have been projected to GRCh37 using UCSC liftover with <1% of features failing to project.

## Enhancing support for genomic interpretation

### A new interface for genome regulation investigation and interpretation

**Figure 1.**
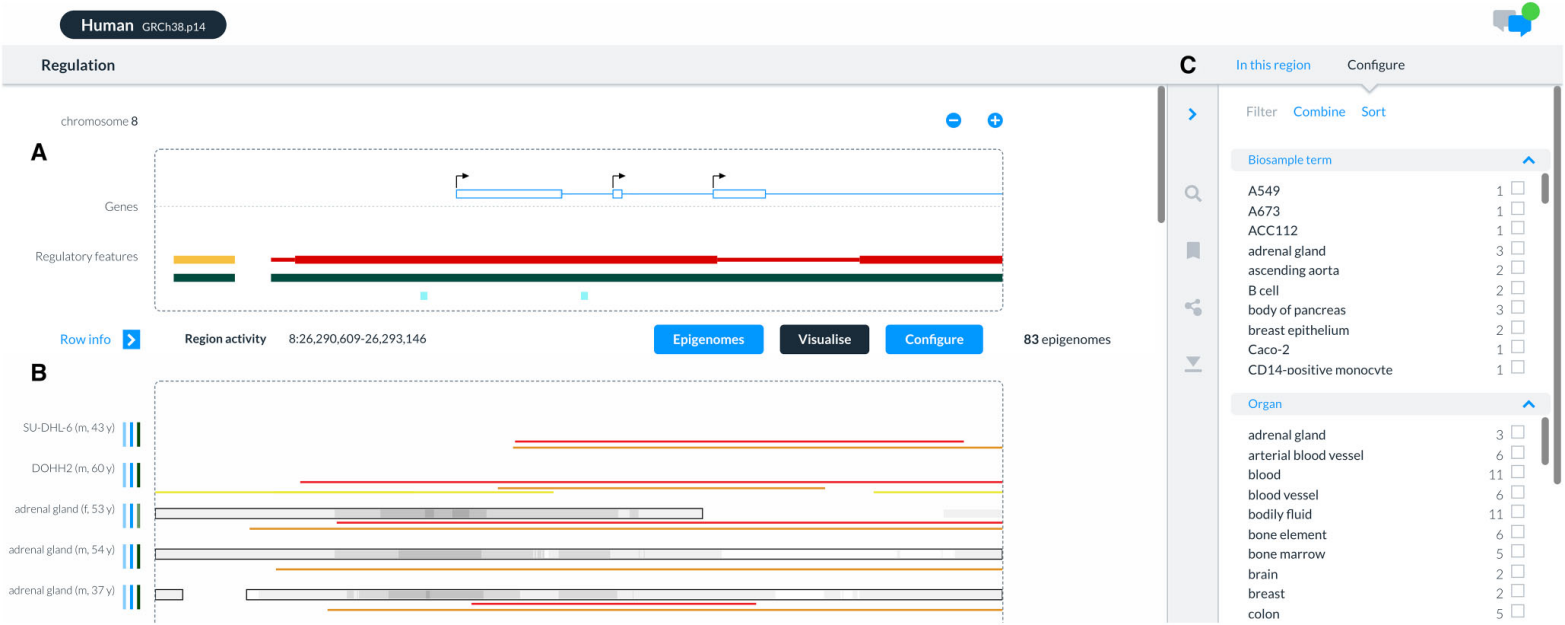
The regulatory activity viewer. (**A**) The gene of interest (PPP2R2A), exon and transcription start sites, and the mapping of regulatory features. Promoters are indicated in red, enhancers yellow, epigenetically modified accessible regions dark green, open chromatin light grey, and CTCF light blue. Each feature can be clicked on to display additional information. The panel can be panned and zoomed by clicking on the + and − buttons. A new focus gene can be selected by clicking on a gene and selecting ‘Make focus’. (**B**) Epigenomic activity across cell/tissue (*y*-axis) with open chromatin and histone marks (*x*-axis). Open chromatin signal is indicated in greyscale, with peaks as black rectangles. Histone mark peaks are shown as lines: H3K4me3 (red), H3K27ac (orange), and H3K4me (lime). Cell/tissue metadata are summarized on the left, with further details available via the ‘Epigenomes’ button. (**C**) Configuration panel displayable by clicking the ‘Configure’ button, offering options to combine or sort epigenomes based on attributes.

Our new visualization (regulation.ensembl.org/115/regulatory_activity) is a reimagining of how to explore regulatory annotation (Fig. 1). We prioritized three enhancements: visualization of signal and peaks across all available epigenomes in the context of gene and regulatory annotation; filtering and sorting epigenomes by key metadata, e.g. life stage, organ; allowing epigenomes to be combined or split based on a dimension of interest, e.g. splitting liver activity by sex or combining into a single liver epigenome. Researchers can choose the attributes to filter displayed epigenomes, combine epigenomes based upon a shared set of attributes, and configure the order of tracks displayed based on the relative importance of selected attributes. The interface is capable of displaying annotations across hundreds of epigenomes within a few seconds. While currently only available for GRCh38, we plan to expand this visualization and roll it out to the remaining nine reference epigenomes (mouse, pig, cattle, chicken, Atlantic salmon, turbot, rainbow trout, common carp, and European seabass) supported by Ensembl in 2026.

### Improving the exploration of epigenome catalogues

The new Ensembl regulation subsite (regulation.ensembl.org) provides a streamlined interface for exploring primary experimental data and sources that underpin our regulatory annotation. It allows researchers to browse annotation, high-level statistics, and key sample metadata sources (including BioSamples, European Nucleotide Archive, and ENCODE) across 3 Ensembl releases and our 10 supported species [36, 37]. Similar to our previously described regulation activity viewer, our regulation subsite allows filtering by multiple attributes, e.g. organ and life stage. The interface gives researchers access to experimental details for each regulatory feature, specifying in which epigenomes it is active or inactive, and the specific experiments and biological replicates used to make that determination.

### Ensembl VEP

The Ensembl VEP, available online via a RESTful API, web tool, and command-line application, has been significantly enhanced with new features for interpreting genetic variants. Ensembl VEP includes NIH All Of Us allele frequency data and new somatic classifications from ClinVar [38, 39]. For human structural variants, we report clinical significance assertions from ClinVar and frequency information from gnomAD on overlapping structural variant sites. Ensembl VEP can indicate when a variant falls within a GENCODE promoter, a key feature for identifying potential non-coding disease associations.

Multiplexed assays of variant effects (MAVEs) from the MaveDB resource measure the effect of all possible variants in a region on a cellular phenotype, which provides insights into possible disease impact when the assay method reflects the gene-disease mechanism [40]. We have imported the latest MaveDB version, which represents a 6.4-fold increase in variants covered to ∼7.7 million. Results from MaveDB are mapped to genomic coordinates to enable annotation of variants via Ensembl VEP with MAVE results [41]. Our online tool has been enhanced by embedding publication links, enabling easier interrogation of results. Finally, we have developed a new Ensembl VEP extension to integrate predictions of likely gene disease mechanism derived from a support vector classification model [42].

### Ensembl beta

In September 2025, we archived Ensembl Rapid Release (rapid-archive.ensembl.org). All data in Rapid Release are now available from Ensembl Beta, and all Rapid URLs redirect to the most appropriate Beta page using the Resolver API (resolver.ensembl.org). The rate of data release has now increased to ∼150 genomes every two weeks. Functionality has been extended to display population frequency data from EVA release 7 [43]. We have also made the first release of our core data model (CDM, https://github.com/Ensembl/ensembl-cdm-docs) and variant data model (VDM, https://github.com/Ensembl/ensembl-vdm-docs), a complete rebuild of the Ensembl data model for gene models, assemblies, metadata, and variant events. These new models drive our GraphQL API (https://beta.ensembl.org/data/graphql), providing a robust and modern foundation for data access and integration.

### Supporting integrated and partial data release

**Figure 2.**
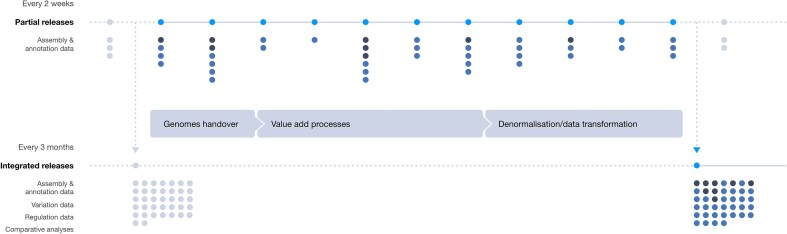
Two release processes are shown executing in parallel. Release points are indicated as light blue circles. Below each release point, new assemblies (dark blue dots) and annotation updates (black dots) are indicated. Genomes that were published prior to the start of an integrated release cycle are collated, and an integrated release is minted. In parallel, partial releases of data are made. After an integrated release is made, the next integrated cycle starts and brings in all partially released data.

The Ensembl project has been historically characterized by stable, versioned, and fully integrated data releases providing consistent datasets that can be cited in long-term analyses and support reproducible research. The time between these release cycles was significant in length, slowing access to new data. By contrast, data made available through our rapid release platform offered timely access to new genomes but with limitations: data were not systematically integrated across the resource and lacked long-term persistence. Ensembl Beta now supports two types of release cycles: an integrated release and a partial release. The integrated release corresponds to the traditional Ensembl release cycle, a fully synchronized update of Ensembl where data are coordinated approximately every 3 to 4 months. The partial releases provide genomes or subsets of annotations without a full integration across Ensembl but provide access to new data every ∼2 to 3 weeks (Fig. 2). In February 2025, the first Ensembl Beta integrated release made 2919 genomes available for long-term use; >1200 genomes were subsequently published in partial releases. Our strategy provides researchers with a choice between stable, consistent datasets or more immediate updates, depending on their requirements.

## Training and support

We continue to offer a comprehensive training programme, delivering 71 in-person and virtual events to 4326 participants over the past year. This year witnessed the launch of our first virtual Train the Trainer (TtT) course to build capacity in bioinformatics education using Ensembl training materials. The initial course was delivered in collaboration with the Kano Independent Research Center Trust in Nigeria with 15 participants. Our second course expanded to a pan-African group combining recorded lectures and assignments to increase accessibility to our materials across 10 countries, reaching 15 participants. In June, our first workshop on using Ensembl Beta was delivered to another pan-African cohort of over 1700 participants across 50 classrooms in collaboration with H3ABioNet and the African Genomics Data Hub. We also piloted our first public engagement activity in Latin America, introducing students to bioinformatics by demonstrating DNA extraction from local fruits and exploring the genomics of regional dish ingredients. Materials from this course are available from our training website in both English and, for the first time, Spanish. Our outreach team is available to deliver courses on all Ensembl platforms, tools, and TtT to both in-person and virtual audiences. Support for Ensembl tools and data is available via our helpdesk and developer mailing list. Our training materials (available under a CC-BY 4.0 license) and calendar of upcoming events are available from https://training.ensembl.org, with additional events available from the EMBL-EBI training portal (https://www.ebi.ac.uk/training/events). Additional information concerning Ensembl can be found on our blog (https://www.ensembl.info), including release announcements and tool guides.

## Future directions

Recently, the EBP has announced it will increase its efforts 10-fold in pace in order to reach its goal of sequencing all 1.67 million known species by 2035 [44]. As such, we recognize a need to accelerate our transition to our new platform. We plan to complete the migration of all genome assemblies and annotations by mid-2026 and have targeted Ensembl 116 (Q2-2026) as the last release on our current platform. All existing Ensembl sites and tools will become archives and will receive only high-priority data patches and infrastructure maintenance for one year. Our probe mapping resources will receive their last update in Ensembl 116 and remain accessible only via our archives. Researchers who work with biodiversity data and human pangenomes and require access to our latest data will be best served through our new infrastructure. Those reliant on our existing whole-genome comparative visualization, existing regulation interfaces, AlphaFold Variant visualization, and functional annotation including phenotype associations, and Gene Ontology annotations should remain on our current infrastructure while these visualizations and data are migrated [45]. Our new infrastructure’s FTP site will provide access to data not yet displayable on our resource. We will develop a new set of interfaces and enhancements, including visualizations of large structural variants and sequence alignments; extend species search to include more filtering facets, including taxonomy, project, and thematic group; and bring our new data warehouse query service online. We will also identify currently available datasets to migrate to the new platform, including support for transcriptomic data and alternative gene models. Progress of the migration to the new infrastructure will be disseminated by our blog and social media channels, which provide advanced announcements concerning major changes when they are released and address frequently asked questions.

## Data Availability

All Ensembl data is made available without restriction from our main website (https://www.ensembl.org), Ensembl Beta (https://beta.ensembl.org) and portals (https://plants.ensembl.org, https://metazoa.ensembl.org, https://fungi.ensembl.org, https://protists.ensembl.org, https://bacteria.ensembl.org, https://grch37.ensembl.org, https://covid-19.ensembl.org). Data is also available for bulk access via our FTP site (https://ftp.ensembl.org) and programmatically (https://rest.ensembl.org,https://beta.ensembl.org/data/graphql). Ensembl code is available from GitHub (https://github.com/Ensembl and https://github.com/EnsemblGenomes) under an open source Apache 2.0 licence. News about our releases and services can be found on our blog (https://www.ensembl.info), our announce mailing list (https://lists.ensembl.org/mailman/listinfo/announce), X (@ensembl; https://x.com/ensembl), LinkedIn (https://www.linkedin.com/company/ensemblgenomebrowser) and Facebook (https://facebook.com/Ensembl.org).
